# The Vitamin D Analogs Doxercalciferol Induces Weight Loss and Improves Fertility in a Mouse Model of Polycystic Ovary Syndrome

**DOI:** 10.1002/rmb2.70082

**Published:** 2026-08-18

**Authors:** Hang‐soo Park, Ana Corachan, Jin Seok, Farzana Liakath Ali, Mohammad Mousaei Ghasroldasht, Safia Akhtar, Hanaa Mohammed, Charlotte Manier, Abdullah Awartani, Nahed Ismail, Hyunho Kim, Ayman Al‐Hendy

**Affiliations:** ^1^ Department of Biomedical Science Sunchon National University Suncheon Republic of Korea; ^2^ Research Institute of Basic Sciences Sunchon National University Suncheon Republic of Korea; ^3^ Department of Obstetrics and Gynecology University of Chicago Chicago Illinois USA; ^4^ IVI Foundation, Instituto de Investigación Sanitaria La Fe Valencia Valencia Spain; ^5^ Human Anatomy and Embryology Department, Faculty of Medicine Sohag University Sohag Egypt; ^6^ Department of Basic Medical Sciences Al Rayan National College of Medicine Madina Saudi Arabia; ^7^ Biological Science Division University of Chicago Chicago Illinois USA; ^8^ College of Medicine King Saud University Riyadh Saudi Arabia; ^9^ Department of Pathology, College of Medicine University of Illinois at Chicago Chicago Illinois USA; ^10^ Dept of Medical Sciences Khalifa University Abu Dhabi UAE

**Keywords:** doxercalciferol, obesity, polycystic ovary syndrome (PCOS), vitamin D analog

## Abstract

**Purpose:**

Polycystic ovary syndrome (PCOS) is the most common endocrine and metabolic disorder in reproductive‐age women. Several published studies demonstrated the therapeutic potential of vitamin D on PCOS condition, but vitamin D supplementation leads to only modest improvements. Doxercalciferol (Dox) is a highly potent vitamin D receptor (VDR) agonist that binds to VDR with at least 150 times greater affinity than other vitamin D compounds. In this study, we hypothesized that the therapeutic potential of Dox may reverse PCOS‐related phenotypes more efficiently.

**Method:**

We first analyzed the regulatory potential of Dox in fat metabolism by comparing adipocyte proliferation and differentiation in our in vitro model. We also injected Dox into our mouse model of PCOS to analyze the therapeutic effect of Dox treatment on various PCOS‐related parameters and fertility.

**Result:**

Dox treatment inhibited the proliferation of both adipose lineage cells and androgen‐producing H295R cells in a dose‐dependent manner. PCOS‐related outcomes such as body weight and fertility rate were significantly reversed in Dox‐treated PCOS mice model.

**Conclusion:**

Our study revealed that vitamin D analogs are a promising family of compounds for reducing body weight and reversing infertility in an animal model of PCOS.

## Graphical abstract

An overview of Doxercalciferol treatment in PCOS mouse model.

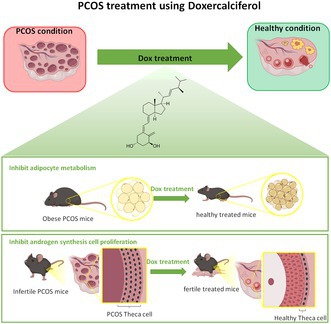

## 1 Introduction

Polycystic ovary syndrome (PCOS) is one of the most common endocrine disorders. Approximately 15%–18% of reproductive‐age women suffer from PCOS [1, 2]. Hyperandrogenism, ovulatory dysfunction, and polycystic ovarian morphology are the major characteristics of PCOS [1]. These symptoms can contribute to anovulation and eventually infertility in PCOS patients [3, 4]. Unfortunately, currently available treatment options are aimed at managing only symptoms. These may include medications such as hormonal contraceptives, insulin sensitizers, and fertility drugs. Lifestyle changes, such as exercise and dietary modifications, can also be beneficial in managing the symptoms of PCOS [5, 6]. However, these methods have not been shown to be effective in addressing the underlying pathogenesis of this condition or restoring fertility in PCOS patients.

The relationships among obesity, hyperandrogenism, and inflammation are important regulatory factors in the pathogenesis of PCOS [7]. According to the published literature, vitamin D treatment has therapeutic potential in patients with PCOS [8, 9, 10, 11] through regulating inflammation [12], and glucose metabolism [13, 14]. These findings demonstrate that other synthetic vitamin D analogs that interact with VDR could be potential candidates for the effective treatment of PCOS through the regulation of metabolic aberrations.

Doxercalciferol (Dox, 1α‐hydroxyvitamin D2) is a highly potent nonhypercalcemic VDR agonist. Dox has been used in several published papers to investigate the protective role of VDR signaling in renal disease models [15, 16]. Recent studies have reported that Dox has multiple effects on pathways involved in renal fatty acid and cholesterol metabolism [15]. Considering these previous studies, herein, we evaluated the therapeutic potential of Dox in both in vitro and in vivo PCOS models. This study is the first to report the therapeutic effect of Dox on PCOS.

## 2 Materials and Methods

### 2.1 Ethics Approval in This Study

The animal experiments were approved by the University of Chicago Animal Care Committee (UC IACUC). The animal protocol entitled “Study for treat female reproductive disease using small animal model” was approved by UC IACUC on 11 March 2020 (approval number 72638). All the animal experiments were performed in compliance with the University of Chicago's policies and guidelines for the use of laboratory animals.

### 2.2 Dox Treatment Conditions

Hectorol Vitamin D (2 μg/mL, Genzyme, Cambridge, MA) Dox was used in this study for both in vitro and in vivo experiments. For in vitro experiments, Dox was added to the cell culture media at concentrations of 1 nM, 10 nM, or 100 nM, and the cells were cultured with the corresponding media to test the effect of Dox. For the in vivo experiment, we intraperitoneally (IP) administered 0.3 μg/kg Dox (100 μL) every other day to mice with PCOS for five weeks. To prepare Dox solution for injection, we diluted 3.5 to 5 μL of Dox solution (2 μg/mL in manufacturer's organic solvent) with PBS to generate the injection volume (100 μL). To prepare the control inject for healthy mice and non‐treated mice, we used 5% DMSO in PBS as the matched vehicle inject solution. The dose of Dox treatment was selected based on our previous study [17, 18].

### 2.3 Cell Culture

The human liposarcoma cell line SW‐872 (Lonza, Basel, Switzerland) was used as an in vitro model of immortalized adipocytes [19, 20]. Human‐adipose‐derived mesenchymal stem cells (MSCs) were purchased from Lonza (Lonza, Basel, Switzerland) and cultured for adipocyte differentiation. Androgen‐producing human adrenocortical carcinoma cells (H295R cells) were used to establish an in vitro model of ovarian theca cells from PCOS patients [21, 22, 23]. The detailed process is described in supplementary data.

### 2.4 Establishment of the Letrozole‐Induced Mouse Model of PCOS

In this study, we used a mouse model of letrozole (LTZ)‐induced PCOS mice model, which we used in our previous publications [24, 25]. Mice were housed no more than 5 mice per cage with free access to food and water. 11 PCOS model mice received vehicle as the PCOS group, and another 11 PCOS model mice received Dox as the treated group (PCOS+Dox). Separately, 11 healthy C57BL6 mice received vehicle as the healthy control group. After treatment, 5 mice per group were sacrificed for analysis, while the other 6 mice per group underwent breeding for further fertility analysis. For the breeding, one male mouse was housed with two female mice. All the animal experiments were performed in compliance with the University of Chicago's policies and guidelines for the use of laboratory animals. Detailed process is described in Table S1.

### 2.5 Glucose Tolerance Test (GTT)

Glucose tolerance testing was performed on mice 5 weeks after Dox treatment. We removed food and allowed the mice to freely access the drinking water in the cage for 16 h (5 p.m. to 9 a.m.) to fast. After fasting, we injected D‐glucose (2.0 g/kg body weight) into the mice via IP injection. Blood glucose levels were measured at 0, 30, 60, 90, and 120 min following glucose injection using a Accu‐Chek Performa (Roche Diagnostics Corp., Indianapolis, IN, USA). Based on the manufacturer's certificate, the coefficient of variation (CV%) for Accu‐Chek blood glucose monitors typically ranges between 2.0% and 3.9%.

### 2.6 Mouse Serum Sample Assay

We collected whole blood (approximately 1 mL per mouse) from all the groups through cardiac puncture under isoflurane anesthesia. We separated the serum (approximately 400 μL per mouse) by centrifugation (2000 × g for 15 min) and stored it at −80°C. Serum hormone levels were measured at the University of Virginia Ligand Core Facility. Serum chemistry was analyzed at the Animal Resource Center at the University of Chicago. The detailed process is described in Table S1.

### 2.7 Tissue Histology Analysis

We collected adipose tissue from the retroperitoneal white fat, ovaries, uterus, kidney, and liver. All tissues were fixed in 4% paraformaldehyde and embedded in paraffin to obtain formalin‐fixed, paraffin‐embedded tissue (FFPE tissue). FFPE tissue sections were stained with hematoxylin–eosin (H&E) for microscopy. The histology core of the University of Chicago (Chicago, IL, USA) performed sample processing, sectioning, and staining. Histological analyses were performed through visual inspection using an Asperio Imaging Scope (Leica Biosystems, Wetzlar, Germany).

### 2.8 White Fat Tissue Adipocyte Size Assay

One piece of retroperitoneal white fat tissue was collected from each mouse and processed to generate FFPE tissue section as described above. The H&E staining images (*n* = 3 per group) were exported into a typical JPEG file for size analysis. We randomly selected 30 adipocytes in each H&E image and measured the size of each adipocyte with Image J software (US National Institute of Health, Bethesda, MD, USA). Then we compared the average size of adipocyte and the size distribution of adipocyte in each group.

### 2.9 Gut Microbiome Analysis

Fresh fecal samples were collected from each mouse for gut microbiome analysis based on the protocol that is described in the published paper [26]. We restrain each mouse by holding the dorsal skin of the mouse between the shoulder and rotating the mouse to make a position that has the ventral side facing up. Through a gentle stroke of the mouse belly until it defecates, we collect fecal samples using sterile forceps and a tube. Each collection was taken within 2 min, and 1 to 2 fecal pellets were collected from each mouse. For fresh fecal sample analysis, fecal samples were aliquoted within 24 h into 1.5 mL freezer vials and stored at −80°C. The samples were analyzed with a DFI MICROBIOME METAGENOMICS FACILITY at the University of Chicago (Chicago, IL, USA). The detailed process is described in Table S1.

### 2.10 Statistics

One‐way ANOVA (data with more than three groups) or multiple unpaired *t*‐tests (data with two groups) were performed using GraphPad Prism 9 (GraphPad Software, San Diego, CA, USA) for statistical comparisons between groups. The data were not normally distributed due to a small sample size (< 30). All the data are presented as the mean ± standard deviation (SD) and median and interquartile range (IQR) information to report non‐normally distributed data. Statistically significant differences between groups are marked with * (*p* < 0.05), ** (*p* < 0.005), *** (*p* < 0.0005), or **** (*p* < 0.0001).

## 3 Results

### 3.1 Effect of Doxercalciferol (Dox) on Adipocyte Metabolism

**FIGURE 1:**
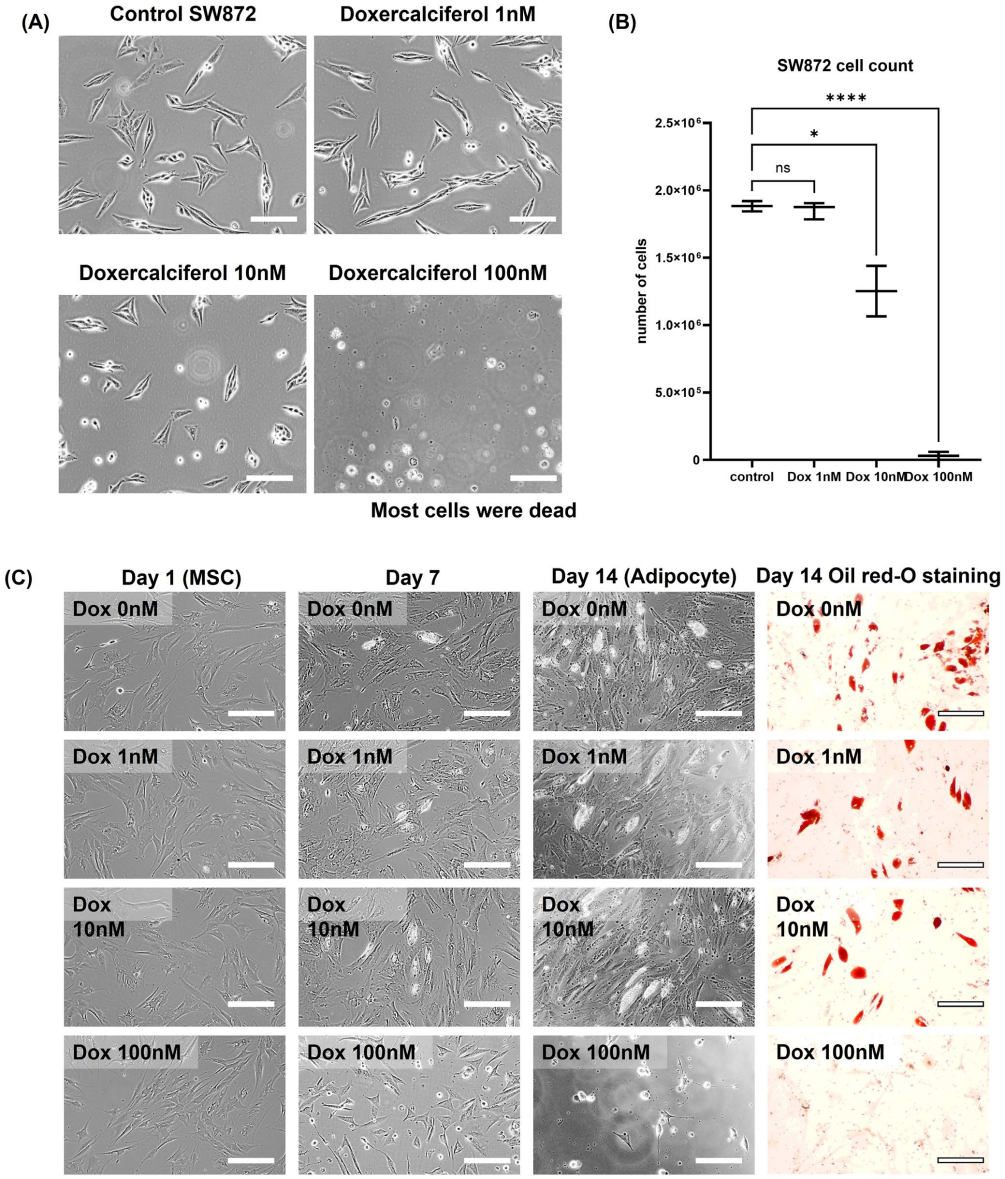
Effect of doxercalciferol treatment on an immortalized adipocyte model (SW872) and MSC‐derived adipocytes. (A) SW872 cell morphology after doxercalciferol treatment (24 h). (B) Comparison of SW872 cell numbers among groups treated with different concentrations of doxercalciferol. (C) Changes in cell morphology after adipodifferentiation (MSC‐derived adipocytes) in response to doxercalciferol treatment. The scale bar size is 250 μm.

We first tested the effect of Dox on adipocyte proliferation using the immortalized adipocyte cell line SW872. We treated cells with 1 nM, 10 nM, or 100 nM Dox for 24 h and counted the cells to determine the proliferation rate. We found that, compared with those in the control group, the number of Dox‐treated cells in the 10 nM group was slightly decreased (*p* = 0.02). Interestingly, most adipocytes did not survive in the 100 nM Dox treatment group, and the number of cells was significantly lower (*p* = 0.001) than that in the control group (Figure 1A,B). We also tested the effect of Dox using mesenchymal stem cell‐derived adipocytes. In undifferentiated MSCs, Dox treatment did not cause toxicity in the tested range (1 to 100 nM). We found that the number of differentiated adipocytes (positive Oil Red O staining) was decreased in the Dox‐treated groups, but there were no differentiated adipocytes in the 100 nM Dox treatment group (Figure 1C).

After we confirmed that Dox suppressed adipocyte proliferation and differentiation, we also analyzed adipogenic gene expression levels in the SW872 cell line. Through the analysis of 84 key genes involved in the differentiation and maintenance of mature adipocytes, we found that several genes were altered by Dox treatment (Figure S1). We found that the majority of the analyzed adipogenesis regulator genes (69 out of 84 genes) exhibited a decreasing trend. Among those altered genes, four adipogenesis regulator genes, PRDM16 (0.59 ± 0.05‐fold), SREBF1 (0.77 ± 0.02‐fold), WNT1 (0.75 ± 0.08‐fold), and WNT10B (0.50 ± 0.09‐fold), were significantly downregulated (*p* < 0.05) after Dox treatment compared with those in untreated control cells. We also found that several adipogenesis regulators, such as CEBPB (2.15 ± 0.43‐fold), CEBPB (1.69 ± 0.19‐fold), and RXRA (1.20 ± 0.04‐fold), were upregulated (*p* < 0.05).

Next, we analyzed adipokine secretion in SW872 cells using conditioned media (Figure S2). Among 62 analyzed cytokines, we found six cytokines were significantly downregulated (*p* < 0.05), such as CXCL10 (0.78 ± 0.12‐fold), MIF (0.72 ± 0.10‐fold), CCL18 (0.77 ± 0.42‐fold), CCL5 (0.86 ± 0.07‐fold), TNF RII (0.61 ± 0.02‐fold), TIMP‐2 (0.65 ± 0.08‐fold), and TNF alpha (0.78 ± 0.09‐fold). Taken together, our data demonstrated that Dox treatment decreases adipogenesis by inhibiting adipocyte proliferation and adipogenic differentiation and downregulating adipogenesis gene and adipokine expression.

### 3.2 Doxercalciferol Inhibits Androgen Production in an in Vitro Model

**FIGURE 2:**
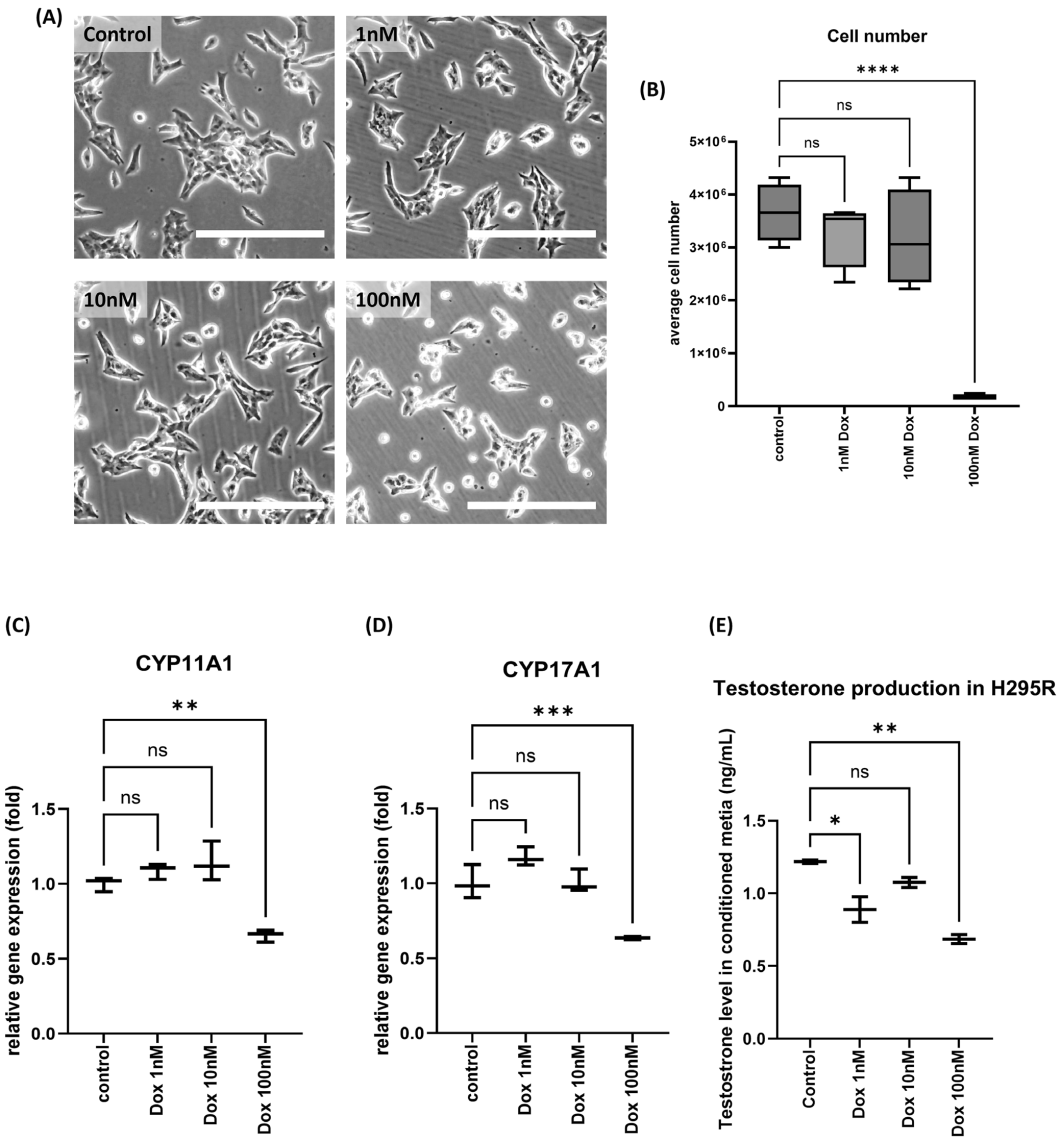
Effect of doxercalciferol treatment on androgen production in H295R cells (theca‐like cells). (A) Cell morphology after treatment with doxercalciferol (24 h). The scale bar size is 250 μm. (B) Comparison of cell numbers in the different doxercalciferol treatment groups. (C) Relative gene expression level of the androgen‐producing gene CYP11A1. (D) Relative gene expression level of the androgen‐producing gene CYP17A1. (E) Testosterone production in H295R cell conditioned media. The data are presented as median and interquartile range (IQR) information to report non‐normally distributed data. (Significance level: *p < 0.05, **p < 0.005, ***p < 0.0005, ****p < 0.0001; NS: Not significant.)

We also tested the effect of Dox treatment on androgen production in our in vitro cell model. We used androgen‐producing H295R cells as an in vitro model. We treated H295R cells with Dox during culture and counted the cells to compare the extent of proliferation (Figure 2A–B). We found that 1 nM and 10 nM Dox treatment did not significantly decrease cell number (*p* = 0.71 and *p* = 0.55, respectively). Interestingly, compared with no treatment (3.66 ± 0.55 × 10^6^), 100 nM Dox treatment significantly decreased (*p* = 0.0001) the number of H295R cells (1.65 ± 0.57 × 10^5^). We also analyzed the expression of androgen‐producing genes, such as CYP11A1 and CYP17A1 (Figure 2C–D). Our RT–PCR data revealed that 100 nM Dox treatment significantly downregulated CYP11A1 (0.66 ± 0.04‐fold, *p* = 0.0015) and CYP17A1 (0.63 ± 0.01‐fold, *p* = 0.0008). We next analyzed the change in testosterone secretion using H295R cell conditioned media. Our ELISA data (Figure 2E) revealed decreased testosterone levels after 100 nM Dox treatment (0.68 ± 0.04‐fold, *p* = 0.004). Taken together, our data demonstrated that Dox treatment decreases androgen production by inhibiting androgen‐producing cell proliferation and downregulating testosterone production.

### 3.3 Doxercalciferol Regulates Metabolism in a Mouse Model of PCOS

**FIGURE 3:**
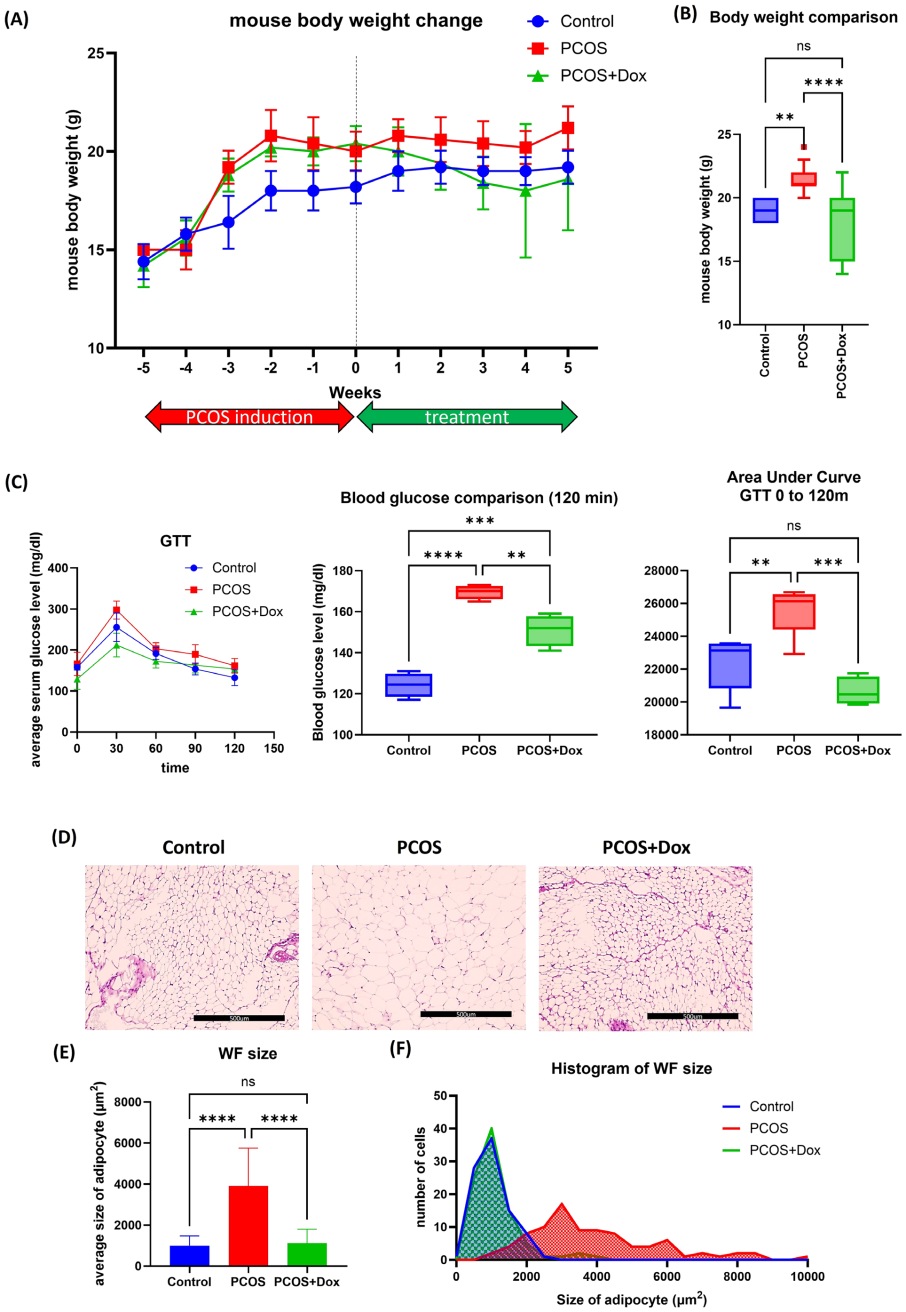
Effect of doxercalciferol treatment on PCOS mice. (A) Mouse body weight change during doxercalciferol treatment in the healthy control group (control), untreated PSOC group (PCOS), and doxercalciferol‐treated PCOS group (PCOS+Dox). The mouse model of PCOS was induced by letrozole for five weeks prior to treatment (week −5 to week 0). During treatment, doxercalciferol was injected (0.3 μg/kg) via the intraperitoneal route every other day for five weeks (weeks 0 to 5). A significant decrease in body weight was observed within two weeks after treatment. (B) Bodyweight comparison between the healthy control group (control), untreated PSOC group (PCOS), and doxercalciferol‐treated PCOS group (PCOS+Dox). (C) Blood glucose levels were compared between groups 120 min after IP‐administered glucose injection. (D) Representative images of white fat tissue. (E) Average size of adipocytes in white fat tissue from control mice, PCOS mice, and PCOS+Dox mice. (F) Histogram of adipocyte size distribution. The scale bar size is 500 μm. The data are presented as the mean ± SD or median and interquartile range (IQR) information to report non‐normally distributed data. (Significance level, *p < 0.05, **p < 0.005, ***p < 0.0005, ****p < 0.0001; NS: Not significant.)

**FIGURE 4:**
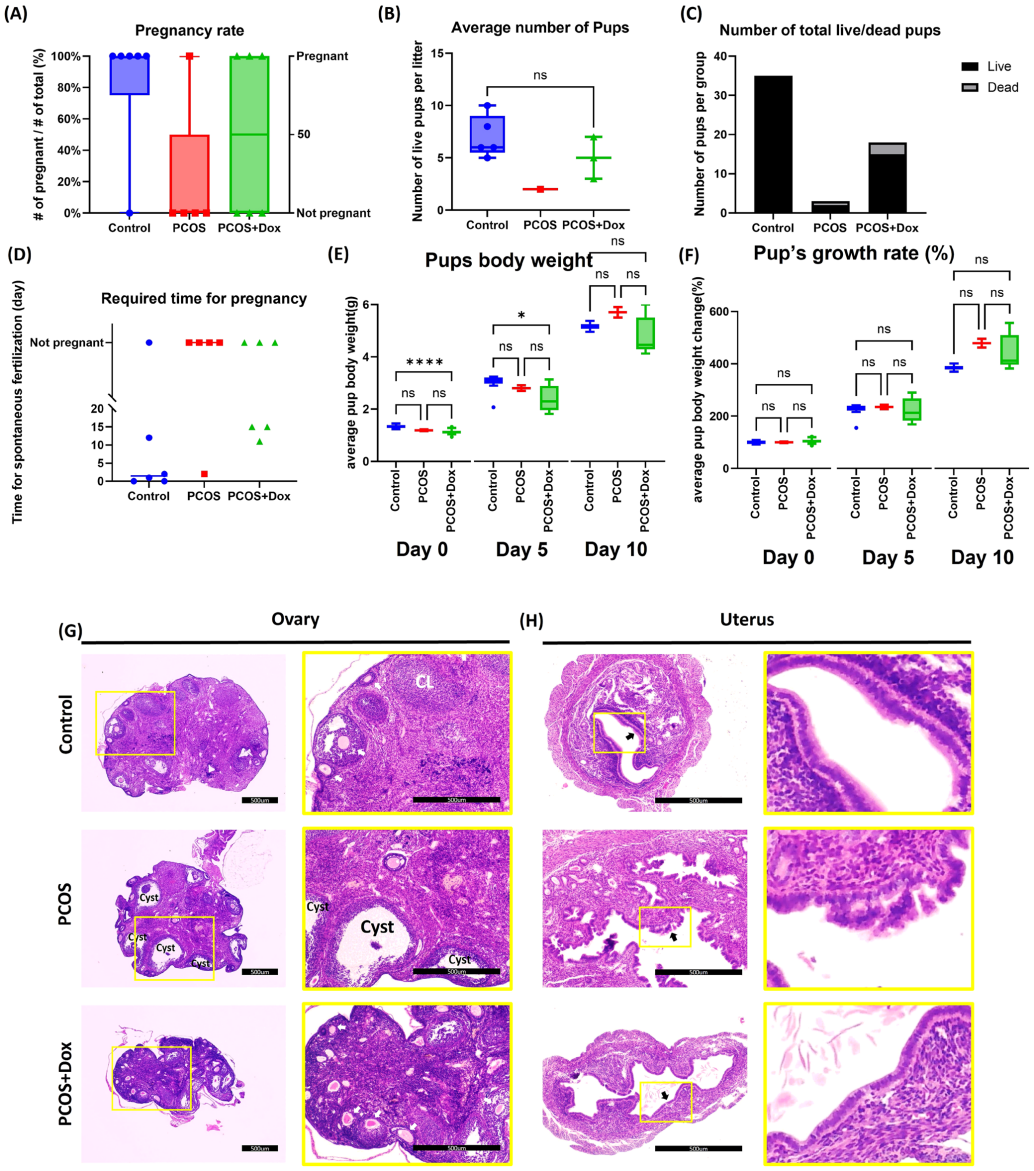
Effect of doxercalciferol treatment on PCOS mouse fertility. Two female mice were bred with one male mouse until pregnant. (A) Pregnant rate of control, PCOS, and PCOS+Dox group. (B) Average number of live pups between groups. (C) Number of total pups per group. (D) Required time for pregnancy between groups. Spontaneous fertilization date was calculated based on delivery date. (E) Average postnatal body weight comparison of pups until day 10. (F) Pups' growth rate (%) comparison until day 10. (G‐H) Female mice were euthanized 5 weeks after doxercalciferol treatment without breeding. Ovary and uterus tissue were collected and fixed in 4% formaldehyde solution for further histological analysis. H&E staining was performed with each tissue section to analyze morphological changes. (G) representing image of ovary tissue with low magnification (left) and high magnification (right). (H) Representing image of uterus tissue with low magnification (left) and high magnification of epithelial region (right). The data are presented as median and interquartile range (IQR) information to report non‐normally distributed data. (Significance level, *p < 0.05, **p < 0.005, ***p < 0.0005, ****p < 0.0001; NS: Not significant.)

We tested the therapeutic effect of Dox in a mouse model of PCOS. We induced a mouse model of PCOS using letrozole, as described in our previous publication. We administered Letrozole via subcutaneous implantation of letrozole pellet (50 μg/day). After 5 weeks of induction, we found some PCOS‐related symptoms in mice model, such as significantly increased body weight (Figure 3A). Additional PCOS parameters, such as hormonal changes, insulin resistance, histological changes, and fertility, were analyzed at the end of in vivo experiment (Figure 3 and Figure 4, Figure S3). We first analyzed metabolic parameters such as body weight change, glucose tolerance, and white fat tissue morphology. Our body weight analysis revealed a clear trend toward a decrease in body weight after Dox treatment (PCOS+Dos), while the body weight of the untreated PCOS mice (PCOS) remained significantly higher (*p* = 0.0031) than that of the healthy control mice (Figure 3A). At 5 weeks after Dox treatment, the body weight of the PCOS+Dox group (17.82 ± 2.68 g) was significantly lower (*p* = 0.0001) than that of the PCOS group (21.55 ± 1.23 g), and there was no difference (*p* = 0.2625) compared with that of the healthy control group (19.00 ± 0.77 g). Next, we analyzed glucose tolerance by measuring blood glucose levels after IP injection of glucose in fasting mice (Figures 3B,C). At 120 min after glucose injection, the average blood glucose level in the PCOS+Dox group was 151.0 ± 7.62 mg/dL, which was significantly lower (*p* = 0.0041) than that in the untreated PCOS group (169.5 ± 3.42 mg/dL). We also collected white fat tissue and analyzed the morphological changes through H&E staining by comparing the average size of adipocytes (Figures 3D–F). We found that adipocyte size was significantly restored after Dox treatment. The average adipocyte size of the PCOS+Dox group mice was 1117 ± 683.9 μm^2^, which was significantly lower (*p* = 0.0001) than that of the untreated PCOS group (3911 ± 1839 μm^2^) and not different (*p* = 0.7953) from that of the healthy control group(1005 ± 469.9 μm^2^) (Figure 3E). Taken together, our in vivo data generated using a mouse model of PCOS suggested that Dox treatment can regulate several metabolic abnormalities in a PCOS model, such as increased body weight, glucose tolerance, and white fat size.

### 3.4 Mitigated Fertility in a Mouse Model of PCOS by Doxercalciferol Treatment

To analyze fertility capacity after Dox treatment, we bred female mice. According to our breeding data, 83.3% of the healthy control mice (5 out of 6) and only 20% of the mice in the PCOS group achieved pregnancy (1 out of 5). Interestingly, compared with untreated PCOS mice, Dox‐treated PCOS mice (PCOS+Dox) had a higher pregnancy rate (50%, 3 out of 6) (Figure 4A–D). A total of 35 live pups and no dead pups were included in the control group. On the other hand, the PCOS group delivered only 2 live pups and one dead pup. Interestingly, the PCOS+Dox group produced 15 live pups and 3 dead pups, which was greater than that in the untreated PCOS group (Figure 4C). We measured the body weight of the pups to compare the growth rate. Although the pups from the PCOS+Dox group were slightly smaller on Day 0 (*p* = 0.0001) and Day 5 (*p* = 0.013), there was no difference on Day 10 (*p* = 0.3506) (Figure 4E).

**FIGURE 5:**
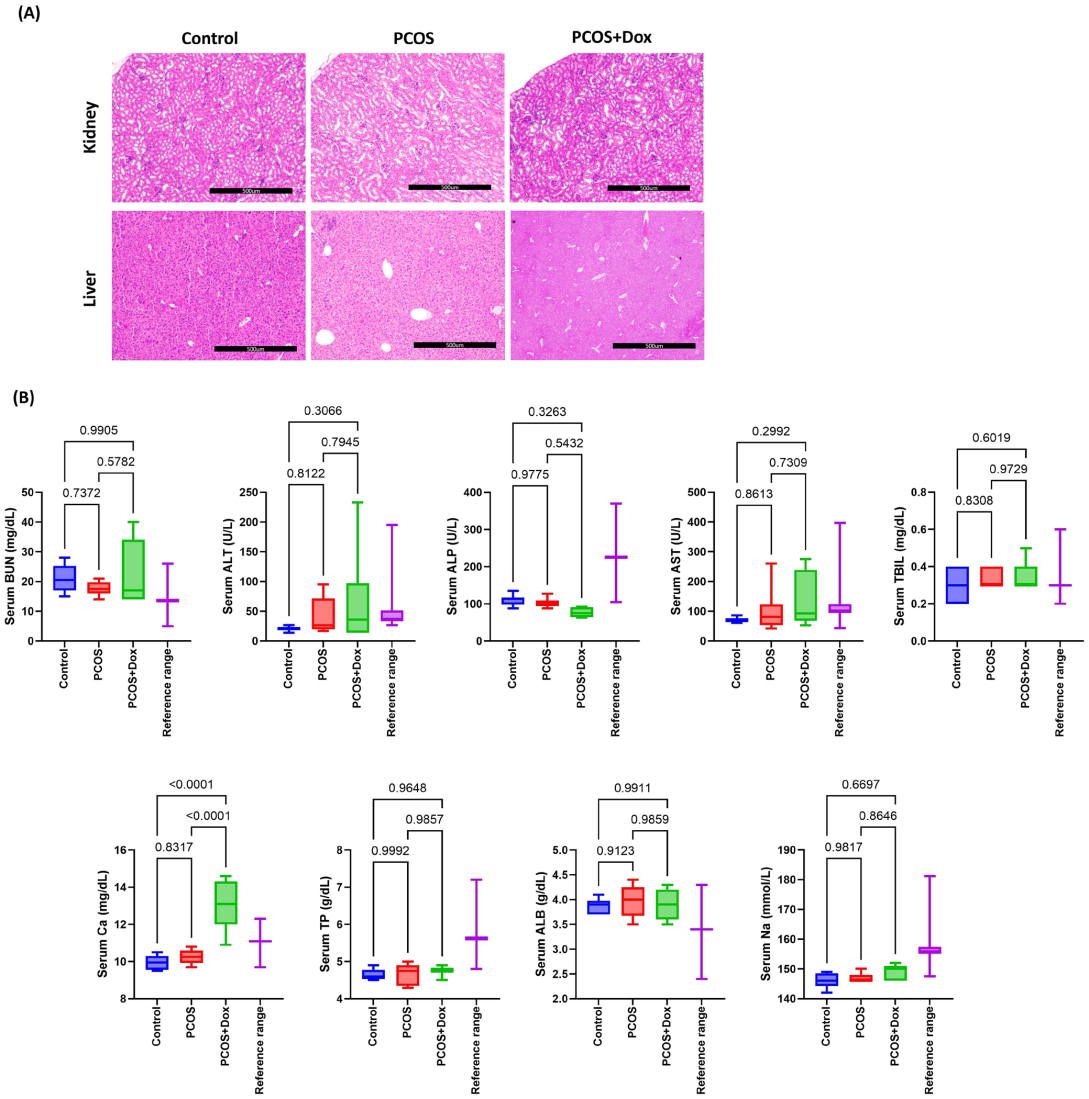
Safety of Doxercalciferol treatment on PCOS mouse tissue toxicity and serum chemistry. (A) Tissue toxicity analysis in liver and kidney. Female mice were euthanized 5 weeks after doxercalciferol treatment without breeding. Kidney and liver tissue were collected and fixed in 4% formaldehyde solution for further histological analysis. H&E staining was performed with each tissue section to analyze morphological changes. Scale bar size is 500 μm. (B) Serum chemistry analysis for BUN, ALT, ALP, AST, TBIL, Ca, TP, ALB, Na+. Each *p*‐value was indicated in pair‐wise comparison. Reference range of C57BL/6 strain was cited from Charles River (Clinical Chemistry Characteristics of Early and Late Adult C57BL/6NCrl mice).

We also analyzed tissue morphology. We first analyzed morphological changes in ovarian and uterine tissue through H&E staining (Figure 4G,H). In untreated PCOS ovaries (PCOS), multiple cystic structures were found instead of healthy follicles. In contrast, Dox‐treated ovaries (PCOS+Dox) had a good number of follicles, similar to that observed in healthy control ovaries (Figure 4G). Our H&E analyses are qualitative observations. Uterus epithelial cells were more organized in the healthy control group and the PCOS+Dox group than in the PCOS group (Figure 4H). We also analyzed further safety parameters such as tissue toxicity and serum chemistry. The tissue toxicity was analyzed by observing the morphology of kidney and liver tissue via visual inspection, and there was no significant evidence of tissue damage or inflammation in either tissue in any of the groups (Figure 5A). To analyze further safety parameters, we analyzed changes in biochemical parameters in serum samples (Figure 5B and Table S1). Our serum chemistry results revealed that most of the parameters were not significantly altered compared with that of control group mice. However, serum calcium level was slightly increased in the Dox treated group (12.9 ± 1.32 mg/dL) compared with control group (9.94 ± 0.37 mg/dL). We also compared our data with the reference range of C57BL/6 strain serum chemistry provided by Lab animal vendor (Charles River, Wilmington, MA, USA) and confirmed that most of the parameters were within the reference range for the healthy C57BL/6 strain. Taken together, our breeding data showed that Dox treatment partially mitigated reproductive dysfunction in a mouse model of PCOS without any side effects.

### 3.5 Doxercalciferol Regulates the Gut Microbiome Profile in a Mouse Model of PCOS3

**FIGURE 6:**
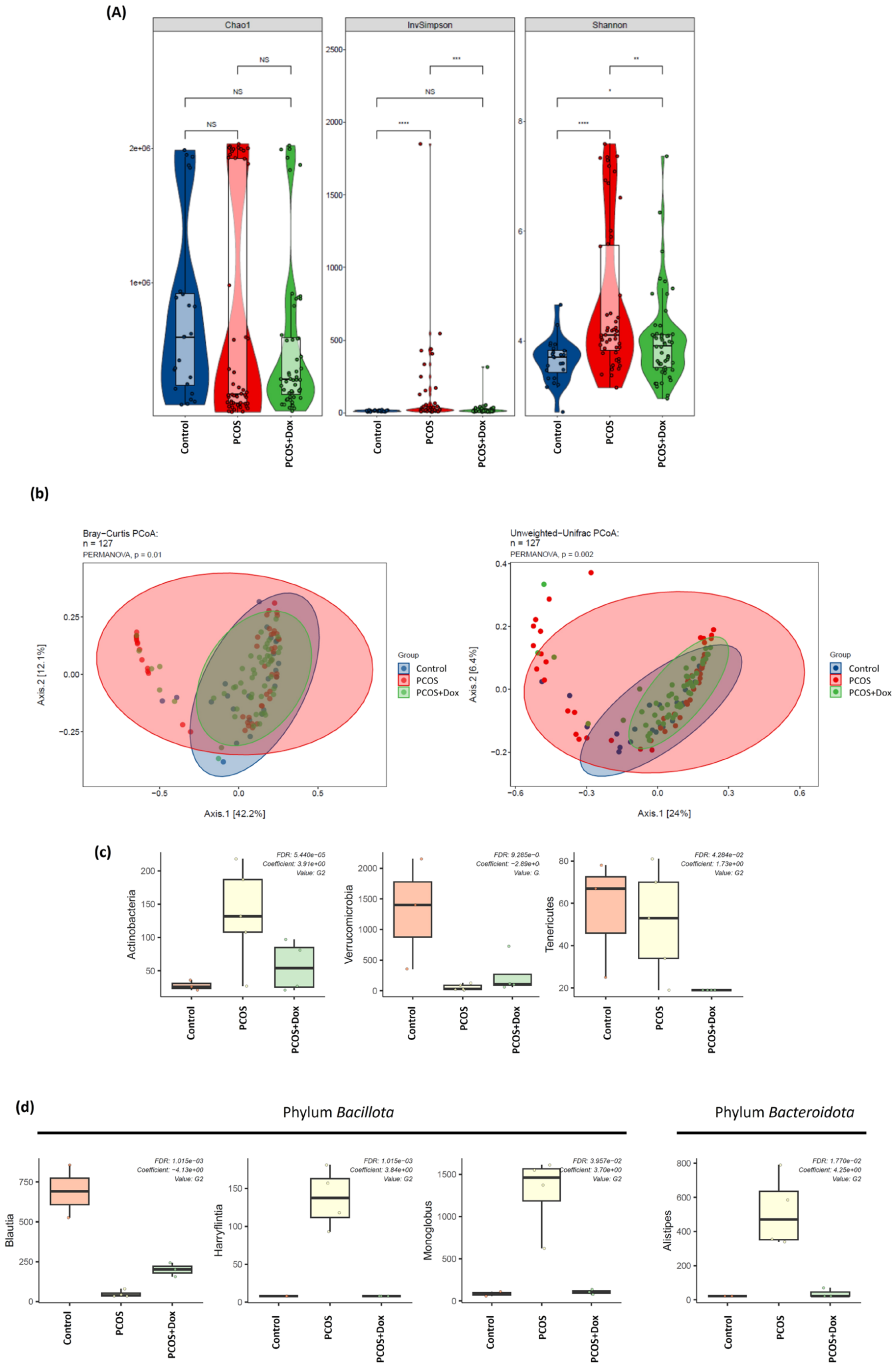
Effect of doxercalciferol treatment on the fecal microbiome of PCOS mice. (a) Alpha diversity analysis of the mouse fecal microbiome population using three different indices. PCOS mice exhibit increased alpha diversity in the fecal microbiome population. (b) Beta diversity analysis of the mouse fecal microbiome population using the Bray–Curtis and UniFrac indices. The PCOS mice exhibited unique microbiome profiles that did not exist in the healthy control or dox‐treated groups. (c) Differential abundance assay at week 10 (MaAsLin2_Phylum level). Compared with healthy controls, PCOS patients had a greater abundance of the phylum Actinobacteria and a lower abundance of the phylum Verrucomicrobia. On the other hand, compared with no treatment, doxercalciferol treatment reduced the abundance of the phyla Actinobacteria and Tenericutes in the PCOS mouse group. The data from other time points (weeks 0–9) were not statistically meaningful due to the low sample number and high diversity. (d) Differential abundance assay at week 9 (MaAsLin2_Genus level). PCOS patients had a decreased abundance of the genus Blautia (phylum Bacillota) and increased abundance of the genera Harryflintia (phylum Bacillota), Monoglobus (phylum Bacillota), and Alistipes (phylum Bacteroidota). On the other hand, these changes in microbiome abundance reversed to healthy control levels after treatment with doxercalciferol. The data from other time points (weeks 0–8) were not statistically meaningful due to the low sample number and high diversity.

Recent studies have reported that the gut microbiome profile changes in PCOS conditions. To further analyze the effects of Dox treatment on the gut microbiome, we analyzed fecal samples from the mice. We collected fresh fecal samples from healthy control mice, PCOS mice, and PCOS+Dox mice and analyzed the microbiome population through 16S rRNA MiSeq sequencing (Figure 6). According to our alpha diversity analysis, all three algorithms (Chao1, InvSimpson, and Shannon) exhibited greater diversity in PCOS mice than in healthy control mice. Interestingly, compared with untreated PCOS mice, Dox‐treated PCOS mice (PCOS+Dox) exhibited decreased diversity, similar to that of control mice (Figure 6A, Figure S4). The beta diversity also showed similar patterns. We analyzed beta diversity through two different algorithms (Bray–Curtis and unweighted UniFrac). The PCOS group exhibited greater diversity than the healthy control group did, and the PCOS+Dox group almost overlapped with the healthy control group (Figure 6B, Figure S5). Through a differential abundance assay at the phylum level, we found that several phyla were altered in the PCOS group and PCOS+Dox group (Figure 6C). We also found some altered microbiome populations at the genus level. According to our differential abundance assay data, the genus Blautia was decreased in the PCOS group compared with the untreated healthy control group and was reversed in the PCOS+Dox group. On the other hand, the genera Harryflintia, Monglobus, and Alistipes were significantly more abundant in the untreated PCOS group than in the healthy control group (Figure 6D). Taken together, our microbiome analysis data showed that Dox treatment regulated an altered microbiome population in the mouse model of PCOS. Our data showed that the microbiome population in the Dox‐treated group was similar to that in the healthy control group.

## 4 Discussion

In this study, we showed that Dox treatment decreased adipogenesis by inhibiting adipocyte proliferation and adipogenic differentiation and downregulating adipogenesis‐related gene and adipokine expression. We also report that Dox treatment reduces inflammatory mediator gene expression in adipose‐like cells and decreases androgen production by inhibiting androgen‐producing cell proliferation and downregulating testosterone production. The in vivo data obtained in a mouse model of PCOS suggested that Dox treatment can regulate several metabolic abnormalities, such as increased body weight, glucose tolerance, and white fat size, in a PCOS model. Our breeding data showed that Dox treatment partially mitigated reproductive dysfunction in a mouse model of PCOS without any side effects. We also analyzed gut microbiome populations, and our data showed that Dox treatment regulated an altered microbiome population in the mouse model of PCOS similar to that in healthy mice.

Dox is indeed a pro‐hormone primarily bioactivated in the liver by 1‐alpha‐hydroxylase enzyme (CYP27B1). Interestingly, according to the literature, it demonstrates that extrahepatic tissues, including adipocytes and steroidogenic cells in the ovary, also express the CYP27B1, which indicates the capability of intracrine conversion of vitamin D precursors into their active forms [27, 28]. Unfortunately, due to a lack of a VDR antagonist blocking experiment, the detailed localized bioactivation mechanism was not clearly confirmed in the current data. This limitation of the current study will be studied in a further study via VDR antagonist blocking experiment.

We used H295R cell line as an in vitro model of ovarian theca cells in PCOS condition based on their strong androgen synthesis. The H295R cell line is widely established in the literature as a robust in vitro surrogate for ovarian theca cells in PCOS models because these cells stably express the complete steroidogenic enzyme cascade, including CYP11A1 and CYP17A1, which are critical for androgen biosynthesis [24, 25, 29, 30]. We analyzed CYP11A1 and CYP17A1 gene expression in Dox‐treated cells as a representative marker of testosterone production. It is well known that the expression of the CYP11A1 gene leads to the synthesis of the mitochondrial cytochrome P450 side‐chain cleavage, which initiates the first step of steroid biosynthesis [31, 32]. The CYP17A1 gene makes an essential enzyme called cytochrome P450c17, which can convert cholesterol derivatives into androgen precursors and eventually contribute to testosterone synthesis [33, 34]. Taken together, the downregulation of CYP11A1 and CYP17A1 gene expression indicates reduced steroidogenesis, and our testosterone level analysis data also support this regulatory mechanism.

To comprehensively evaluate the effects of Dox in an animal model, the optimal study design would include a healthy control group treated with Dox, alongside multiple experimental groups receiving various dosages. However, due to the limited resources, we were unable to include a Dox‐only group or multiple dose–response groups in this study. Nevertheless, the safety profile of Dox has been well reported in both pre‐clinical and clinical studies [35, 36, 37, 38]. For instance, Choi et al. [35] reported the absence of significant adverse effects, such as elevated serum calcium levels, in healthy rats following Dox administration. Furthermore, several clinical trials [36, 37] have demonstrated a low incidence of severe adverse events including hypercalcemia, and those events were restored rapidly upon cessation of treatment. In addition, Choi et al. [35]. also reported that approximately 1 μg/kg of Dox injection was not inducing any significant difference compared with vehicle injection in rat model, although it was much higher dosage compared with our experimental design. Based on these reports, we considered Dox as a sufficiently safe reagent that would not induce severe adverse physiological changes in the healthy mice within our experimental design and selected a single Dox treatment dosage based on our previous study [17, 18] to use our limited resource effectively. While this rationale supports our experimental setup, we acknowledge that the absence of the Dox‐only control remains a limitation in interpreting our data. The primary objective of this study was to establish the therapeutic potential of Dox for the PCOS, deferring dose optimization to subsequent research. To determine the most effective dosage in the PCOS model, future studies will incorporate multiple dose–response groups as well as a Dox‐only control. These subsequent investigations will enable a comprehensive evaluation of both the optimal therapeutic dosage for PCOS and any potential side effects in healthy animals.

In this study, we used a letrozole‐induced PCOS mouse model as our disease model. Letrozole, an aromatase inhibitor, is widely used for PCOS induction, as this model comprehensively recapitulates both the reproductive and metabolic phenotypes characteristic of the PCOS [24, 39]. Alternatively, dehydroepiandrosterone (DHEA) or dihydrotestosterone (DHT) can be used to establish PCOS models in rodents [40, 41, 42]. Furthermore, high‐fat, high‐sugar diets have been utilized to induce PCOS‐like conditions through altered metabolism and diet‐induced obesity [43, 44, 45]. Additionally, the co‐administration of letrozole and a high‐fat diet has been shown to successfully induce PCOS with clear insulin resistance in rat models [46]. Given the distinct advantages and limitations of each model, recent literature indicates that the letrozole‐induced model is particularly advantageous for studying metabolic disturbances, whereas the combination of letrozole and a high‐fat diet is optimal for investigating PCOS‐associated metabolic abnormalities and gut microbiota alterations. Conversely, testosterone administration is deemed more appropriate for evaluating the reproductive abnormalities of PCOS [47]. We have previously published multiple studies using the letrozole‐induced model, consistently demonstrating its efficacy in manifesting key PCOS‐related phenotypes, including anovulation, insulin resistance, and altered lipid metabolism [24, 25]. While we relied on this established mouse model for the current investigation, we acknowledge that it is an artificial disease model with inherent limitations, including potential batch‐to‐batch and animal‐to‐animal variations during model induction. In addition, none of these induced PCOS mouse models including DHEA or letrozole, PCOS‐like phenotypes are generally reversible upon treatment withdrawal [48, 49, 50, 51] which make difficult to perform breeding without effect of model inducing reagent. Moreover, due to the limited time and resources we have presented representative images suggest an ameliorative trend in this study. Future quantitative validation is required to confirm the restoration of ovarian architecture. For future research, it will be imperative to investigate the therapeutic efficacy of Dox across diverse PCOS models, including diet‐induced and DHEA/DHT‐induced models, and enough sample size to validate and expand upon our current findings.

One of the main conditions associated with PCOS is obesity, and previous studies have indicated a strong association between the gut microbiome composition and sex hormones. The intestinal microbiome has dual interactions with PCOS, and excess androgen during the prenatal period reportedly results in gut microbial dysbiosis [52, 53]. An altered composition of the gut microbiota, including diverse bacterial populations and the presence of specific genera, has been observed between control and PCOS conditions both in clinical studies and in animal models [54, 55, 56]. On the basis of our data, we also found similar microbiome changes. Therefore, our microbiome analysis data indicate that the gut microbiome is regulated in PCOS conditions through Dox treatment and that the use of Dox is another benefit of PCOS treatment.

PCOS is a devastating condition that has major negative physical and emotional effects on women. The need for a novel scientific therapy option for women with PCOS is evident. Traditional PCOS treatment such as oral contraceptive pills (OCPs) typically target patient symptoms and fertility needs. Unfortunately, none of the available treatment options provide an effective remedy or directly address the underlying pathogenesis of this condition [57]. For patients seeking fertility, ovulation induction with oral fertility medications and assisted reproductive techniques are the mainstay therapies, but they have limited success and potential major side effects, including life‐threatening complications such as gonadotropin‐induced ovarian hyperstimulation syndrome [58].

Due to the complicated regulatory pathways involved in obesity and fat metabolism, it is not easy to determine a simple and solid explanation of the mechanism involved in Dox treatment. The published literature also illustrates the complex relationship between multiple pathway activities and white fat tissue mass [59]. The simple linear changes in white fat and body weight may not be simple at the molecular level. This topic might be interesting for our future studies to identify the regulatory pathway of Dox treatment.

According to our in vitro adipogenesis gene expression analysis we found the expression level of many adipogenesis regulators decreased after Dox treatment. However, the expression patterns of several adipogenesis genes have shown controversial results. For example, one of the significantly downregulated genes, PRDM16, is known as a pro‐brown adipocyte regulator and is reported to increase insulin sensitivity [60]. The upregulated genes and pro‐adipogenesis regulator CEBPB/CEBPD are also reported to increase lipid metabolism and insulin resistance [61]. Although there are some controversial results concerning gene expression, our other in vitro and in vivo data still demonstrate that Dox treatment can be an effective medication for controlling PCOS‐induced obesity by inhibiting adipocyte differentiation, reducing white fat adipocyte size, and decreasing body weight. Especially, our in vivo data indicate the significant loss of peritoneal fat by Dox treatment. In human PCOS patients, it is reported that visceral and abdominal fat tissue are frequently increased compared with control group [62]. Taken together, the decreased peritoneal fat in Dox treated PCOS mice demonstrates the clinical potential of Dox to treat common PCOS‐related obesity phenotypes.

To perform the GTT assay, we fast mice through overnight (16 h) prior to glucose administration. Overnight fasting has been considered a standard procedure, widely employed in metabolic studies to establish stable baseline blood glucose levels with minimal intra‐group variation [63]. A published study reported that approximately 60% of the 353 analyzed studies utilized an overnight fasting protocol in their GTT assay between 2018 and 2019 [64]. Consequently, we adopted this conventional approach, overnight fasting, for our experimental design. However, emerging evidence suggests that prolonged fasting in mice raises significant animal welfare concerns, leading to substantial weight loss and hypoglycemia, thereby inducing a physiological state more analogous to starvation in humans [63, 64]. Furthermore, it has been proposed that a shorter fasting duration of 5–6 h, rather than 16–18 h in mice, provides a more accurate translational comparison to human clinical data. In humans, an overnight fast achieves a basal steady state for blood glucose and insulin. However, due to the nocturnal feeding behavior of mice, an overnight fast imposes severe metabolic stress rather than a routine physiological baseline [65]. Additionally, extended fasting has been directly correlated with profound hepatic glycogen depletion, significant mass reduction of muscle, and blunted insulin responsiveness [66]. Taken together, these recent findings indicate that the GTT data obtained following an overnight fasting protocol in this study may be influenced by atypical glycogen depletion and stress‐induced hormonal fluctuations. As recent protocols increasingly advocate for shorter fasting intervals [67], future studies should adopt updated guidelines—such as a 6‐h daytime fast—to minimize both ethical and practical concerns, thereby ensuring the generation of more physiologically relevant and accurate data.

Through the published research, it is reported that Vitamin D can regulate fatty acid synthesis, promote fatty acid oxidation, further reducing lipid accumulation, which eventually reverse the PCOS related symptoms [49, 50]. In addition, another study also reported that Vitamin D can reverse PCOS condition via inhibiting NF‐ĸB which reduces the production of free radicals and pro‐inflammatory cytokines [51]. Dox is a synthetic VDR agonist which may activate the same pathway as that reported for Vitamin D. Therefore, we expected that Dox treatment could reverse PCOS condition through normalized fat metabolism. We prove our expectations through an in vitro differentiation study, and our results showed that MSCs were not differentiated into adipocytes in 100 nM Dox treatment condition. Interestingly, published research reported no cytotoxicity at 100 nM Dox treatment [48]. Taken together, our in vitro data indicates the inhibition of adipogenesis, not cytotoxicity. Another outcome, such as the changes in microbiome population that we reported in this study, could be a consequence of metabolic changes in mice, but the detailed mechanism should be studied in further research.

Some researchers may be concerned about hypercalcemia due to vitamin D or other VDR agonists. Although Dox is reported that there are no clinically significant differences in serum calcium level to incidence of hypercalcemia after treatment [38], another study argued that it has not demonstrated a lower incidence of hypercalcemia [68]. These controversial reports may arise the concern of clinical use of Dox in PCOS treatment, but more recent clinical studies strongly supported the safety of Dox treatment. One study reported that after vitamin D analog treatment including Dox, they observed short‐term changes in calcium, but did not observe any distinctions in calcium between groups at 7–12 months of follow‐up [69]. More recent clinical study reported that the safety profile of Dox was generally favorable, and only 8.9% of patients experienced severe hypercalcemia which exceeds 2.8 mmol/L of serum calcium level [37]. In our data, we also observed slightly increased serum calcium level; however, this mild increasing trend in serum calcium level can be neutralized by consuming calcium reducing supplements such as spirulina supplementation [70]. Although there is no solid data yet, reducing the risk of hypercalcemia using another oral supplement might be an interesting topic for enhancing the safety of Dox treatment. Taken together, there is a possibility of mild serum calcium level change; still, Dox treatment can be considered as a safe treatment option to reverse PCOS condition.

Our study evaluated the therapeutic effect of VDR agonists, such as Dox, for the treatment of PCOS. Our results suggest that vitamin D analog doxercalciferol shows a therapeutic potential for PCOS treatment in a mouse model and a potential therapeutic candidate requiring further target‐specific validation for PCOS patients. These preclinical data demonstrate the expanded application of FDA‐approved medication and a potential treatment option for women with PCOS, as well as guiding the further clinical trial.

## Funding

This study was supported by this study was financially supported by start‐up Fund from the University of Chicago (AA), Sunchon National University Glocal University Fund in 2025 (HP), and National Research Foundation of Korea (NRF) grant RS‐2026‐25476186 (HP).

## Ethics Statement

The animal experiments were approved by the University of Chicago Animal Care Committee (UC IACUC). The animal protocol titled “Study for treating female reproductive disease using a small animal model” was approved by the UC IACUC on 11 March 2020 (approval number 72638). All the animal experiments were performed in compliance with the University of Chicago's policies and guidelines for the use of laboratory animals.

## Conflicts of Interest

The authors declare no conflicts of interest.

## Supporting information


**Table S1:** Effect of doxercalciferol treatment on PCOS mouse serum chemistry.
**Figure S1:** Effect of doxercalciferol treatment on the expression of the immortalized adipocyte model (SW872) gene. (A) Altered gene expression in SW872 cells after doxercalciferol treatment (24 h). (B‐E) Significantly downregulated genes in SW872 cells after doxercalciferol treatment. (F‐H) Genes significantly upregulated in SW872 cells after doxercalciferol treatment. The data are presented as median and interquartile range (IQR) information to report non‐normally distributed data. Unpaired T tests were performed for statistical comparisons between groups. (Significance level, *p < 0.05, **p < 0.005, ***p < 0.0005, ****p < 0.0001; NS: not significant).
**Figure S2:** Effect of doxercalciferol treatment on cytokine secretion in an immortalized adipocyte model (SW872). (A) Antibody‐based obesity‐related cytokine analysis of SW872 secretome data after doxercalciferol treatment (24 h) (B‐H) Cytokine production was significantly downregulated after treatment with doxercalciferol. The data are presented as median and interquartile range (IQR) information to report non‐normally distributed data. Unpaired T tests were performed for statistical comparisons between groups. (Significance level, *p < 0.05, **p < 0.005, ***p < 0.0005, ****p < 0.0001; NS: not significant).
**Figure S3:** Serum hormone analysis of PCOS mice.
**Figure S4:** Weekly‐basis alpha diversity analysis of the fecal microbiome of PCOS mice.
**Figure S5:** Weekly‐based beta diversity analysis of the PCOS mouse fecal microbiome.

## Data Availability

All data are present in the paper and/or the Table S1. Additional data are available from the corresponding author on request.
