# Supplementary material for: The Vitamin D Analogs Doxercalciferol Induces Weight Loss and Improves Fertility in a Mouse Model of Polycystic Ovary Syndrome
Source: Reprod Med Biol. 2026 Aug 18;25(1):e70082. doi: 10.1002/rmb2.70082 (PMC13482908; doi:10.1002/rmb2.70082)
Supplement: Supplementary file 1 [file RMB2-25-e70082-s001.docx]

**Supplementary Materials and Methods**

Adipocyte line culture and adipocyte differentiation

The human liposarcoma cell line SW-872 (Lonza, Basel, Switzerland) was used as an in vitro model of immortalized adipocytes(Nair and Al-Hendy, 2011; Nair, Nguyen *et al.*, 2013). The cells were cultured in DMEM/F12 supplemented with 10% FBS and 1% penicillin and streptomycin (Invitrogen, Carlsbad, CA, USA). Once the cells reached approximately 80% confluence, the cells were trypsinized using the CTS TrypLE Select Enzyme (Gibco, Waltham, MA, USA) and serially expanded. Human-adipose-derived mesenchymal stem cells (MSCs) were purchased from Lonza (Lonza, Basel, Switzerland) and cultured according to the manufacturer’s instructions. MSCs were differentiated into adipocytes using a Mesenchymal Adipogenesis Kit (Sigma, St. Louis, MO) following the manufacturer’s instructions. Lipid vacuoles in the differentiated adipocytes were stained with Oil Red O solution (red).

Androgen-producing cell line culture

Androgen-producing human adrenocortical carcinoma cells (H295R cells) were used to establish an in vitro model of ovarian theca cells from PCOS patients. This immortalized cell line has been used in multiple published papers for analyzing androgen synthesis pathways(Hecker, Newsted *et al.*, 2006; Marti, Bouchoucha *et al.*, 2017; McAllister, Han *et al.*, 2019). We purchased H295R cells from ATCC (Manassas, VA, USA; cat. no. ATCC® CRL-2128™). T75 cell culture flasks were precoated with attachment factor (Gibco, Billings, MT, USA; cat. no. S-006-100), and approximately ^1×106^ H295R cells were cultured in precoated T75 flasks with matched cell culture media. DMEM/F12 (Gibco, cat. no. 21041025), 2.5% Nu-Serum (Corning, Corning, NY, USA), and 1% ITS+ Premix supplement (Corning) were used for the cell culture media. Once the cells reached approximately 80% confluence, the cells were trypsinized using the CTS TrypLE Select Enzyme (Gibco, Waltham, MA, USA) and subsequently serially expanded.

Real-time PCR

RNA was extracted from SW872 and H295R cells via the TRIzol method. Approximately 200,000 cells were incubated with 500 μL of TRIzol (Thermo Fisher Scientific, Waltham, MA, USA). The RNA was purified following the manufacturer’s protocol. After RNA purification, 500 ng of RNA was used for cDNA conversion. The expression of adipogenesis markers was analyzed with SW872 cell cDNA using an RT2 profiler PCR array kit (QIAGEN, Hilden, Germany) and a CFX96 PCR instrument (Bio-Rad, Hercules, CA, USA). Four nanograms of cDNA and 12.5 μL of Universal SYBR Green Supermix (Bio-Rad, Hercules, CA, USA) were added to each well for a total of 25 μL of reaction. PCR was performed according to the manufacturer’s protocol. Gene expression levels were analyzed by the delta-delta CT method. The expression of androgen synthesis marker genes was analyzed with H295R cell cDNA using matched primers. The primers used in this study were as follows: CYP11A1 (forward 5’-GAGGGAGACGGGCACACA-3’; reverse 5’-TGACATAAACCGACTCCACGTT-3’; CYP17A1 (forward 5’- GGCCTCAAATGGCAACTCTAGA-3’; reverse 5’-CTTCTGATCGCCATCCTTGAA-3’; and GAPDH (forward 5’-TGACATCAAGAAGGTGGTGAAGC-3’; reverse 5’- CCCTGTTGCTGTAGCCGTATTC-3’. Quantitative real-time PCR (qPCR) was performed using a CFX96 PCR instrument and SYBR Green Supermix (Bio-Rad, Hercules, CA, USA) in a 20-μL final reaction volume. Gene expression levels were analyzed by the delta-delta CT method.

Semiquantitative cytokine array

To collect the SW872-conditioned media, the cells were cultured for 24 h in serum-free DMEM/F12 (Gibco). After incubating for 24 h, we collected the conditioned media and centrifuged it for 5 min at 500 ×g and 4 °C to remove the cell debris. The levels of cytokines in the SW872-conditioned media were analyzed via a membrane-based antibody array (Ray Biotech, GA, USA) following the manufacturer’s protocol. The membranes were visualized using a Chemi Doc XRS +molecular imager (Bio-Rad, Hercules, CA, USA). The signal density of each protein band was quantified using ImageJ software (US National Institute of Health, Bethesda, MD, USA) and normalized to that of the internal control.

ELISA

To collect H295R-conditioned media, cells were cultured for 24 h in DMEM/F12 (Gibco) serum-free media. After incubating for 24 h, we collected the conditioned media and centrifuged it for 5 min at 500 ×g and 4 °C to remove the cell debris. Testosterone in the H295R conditioned media was measured with a Testosterone Parameter Assay Kit (R&D Systems, Minneapolis, MN) following the manufacturer’s instructions.

Establishment of the letrozole-induced mouse model of PCOS

In this study, we used a mouse model of letrozole (LTZ)-induced PCO, which we used in our previous publications(Chugh, Park *et al.*, 2021; Park, Cetin *et al.*, 2023). The animal experiments were approved by the University of Chicago Animal Care Committee (UC IACUC). The animal protocol titled “Study for treating female reproductive disease using a small animal model” was approved by the UC IACUC on 11 March 2020 (approval number 72638). All the animal experiments were performed in compliance with the University of Chicago’s policies and guidelines for the use of laboratory animals. Three-week-old female C57BL/6 mice were purchased from Charles River (Wilmington, MA, USA) for the animal experiment. Mice were housed in an animal facility for at least 72 h under specific pathogen-free conditions. At 3.5 weeks of age, a placebo or 5 mg of LTZ pellet (Innovative Research of America, Sarasota, FL, USA), which released LTZ constantly (50 μg/day) for 90 days, was subcutaneously implanted into the mice (n = 11/group). The healthy control group mice received a placebo instead of LTZ, and body weight was monitored on a weekly basis. Body weight changes were used to monitor the development of PCOS characteristics. Five weeks later, the mice treated with LTZ developed PCOS. After PCOS induction, we intraperitoneally (IP) injected 0.3 µg/kg Dox every other day into mice with PCOS for five weeks. For further analysis, the mice were divided into breeding cages (n = 6 per group) and experimental cages (n = 5 per group). After recovery, the breeding cage mice started breeding with male mice (two females with one male). The experimental mice were subjected to a glucose tolerance test (GTT) five weeks after Dox treatment and were anesthetized before the serum, white fat tissue, ovaries, uterus, kidney, and liver were collected. Tissues were fixed in 4% paraformaldehyde and embedded in paraffin for histological analysis.

Glucose tolerance test (GTT)

Glucose tolerance testing was performed on mice 5 weeks after Dox treatment. We removed food and allowed the mice to freely access the drinking water in the cage for 16 h (5 p.m. to 9 a.m.) to fast. After fasting, we injected D-glucose (2.0 g/kg body weight) into the mice via IP injection. Blood glucose levels were measured at 0, 30, 60, 90, and 120 min following glucose injection using a Bayer glucose monitor (Roche Diagnostics Corp., Indianapolis, IN, USA).

Mouse serum sample assay

We collected whole blood (approximately 1 ml per mouse) from all the groups through cardiac puncture under isoflurane anesthesia. We separated the serum (approximately 400 μL per mouse) by centrifugation (2000 × g for 15 min) and stored it at −80 °C. Serum hormone levels were measured at the University of Virginia Ligand Core Facility. We analyzed the serum testosterone (T), AMH, and insulin levels using ELISA. Serum chemistry was analyzed at the Animal Resource Center at the University of Chicago using a VETSCAN® VS2 Chemistry Analyzer (Zoetis, Parsippany-Troy Hills, NJ). ALB, ALP, ALT, AST, BUN, Ca, Cl-, CRE, GLOB, GLU, K+, Na+, TBIL, tCO2, and TP were measured and compared with control group. The reference range of the healthy C57BL/6 mouse strain (provided by Charles River) also displayed together.

Gut microbiome analysis

Fresh fecal samples were collected from each mouse for gut microbiome analysis. Fecal samples were aliquoted within 24 hours into 1.5 ml freezer vials and stored at -80 °C. The samples were analyzed with a DFI MICROBIOME METAGENOMICS FACILITY at the University of Chicago (Chicago, IL, USA). For 16S rRNA amplicon sequencing analysis, DNA was extracted from fecal samples using the QIAamp Power Fecal Pro DNA Kit (QIAGEN, Hilden, Germany), and the V4-V5 region of the 16S rRNA genes was PCR amplified using barcoded dual-index primers. Illumina compatible libraries were generated using the Qiagen QIASeq 1-step amplicon kit (QIAGEN, Hilden, Germany), and sequencing was performed on the Illumina MiSeq platform (Illumina, San Diego, CA) in the Functional Genomics Facility at UChicago using 2×250 paired-end reads, generating 5,000-10,000 reads per sample. We analyzed 129 samples, and two samples were removed during the quality filtering stage of our pipeline. We used dada2 (v1.18.0) as our default pipeline for processing MiSeq 16S rRNA reads, with minor modifications, in R (v4.0.3). Specifically, reads were first trimmed to 190 bp for both forward and reverse reads to remove low-quality nucleotides. Chimeras were detected and removed using the default consensus method in the dada2 pipeline. ASVs with lengths between 320 bp and 365 bp were retained and deemed high-quality ASVs. The taxonomies of the resultant ASVs were assigned to the genus level using the RDP classifier (v2.13) with a minimum bootstrap confidence score of 80. Species-level classification was performed using BLASTN (v2.13.0) and the RefSeq_RNA database (updated 2022-06-10).

**Ethics approval and consent to participate:** The animal experiments were approved by the University of Chicago Animal Care Committee (UC IACUC). The animal protocol titled “Study for treating female reproductive disease using a small animal model” was approved by the UC IACUC on 11 March 2020 (approval number 72638). All the animal experiments were performed in compliance with the University of Chicago’s policies and guidelines for the use of laboratory animals.

**Authors' contributions:** H. Park developed the experimental design, performed the experiments, analyzed the data, and wrote the manuscript as the first author. All the other authors contributed to the manuscript by writing some paragraphs, searching the literature, and editing. H. Park and A. Corachan designed the in vitro study for the adipogenesis model. H. Park, A. Corachan and J. Seok performed the in vitro and in vivo studies. F. Liakath Ali and M. Ghasroldasht contributed to the data analysis and manuscript writing. S. Akhtar and O. Samour contributed to the animal tissue collection and preparation. H. Mohammed, C. Manier, and A. Awartani contributed to the animal sample collection and literature search for manuscript writing. N. Ismail contributed microbiome analysis data. A. Al-Hendy led the entire study as the corresponding author. All the authors read and approved the final manuscript.

**Data and materials availability:** All the data are presented in the paper and/or the supplementary materials. Additional data are available from the corresponding author upon request.

**Supplementary Table**

Table S1. Effect of doxercalciferol treatment on PCOS mouse serum chemistry.

| **Mouse  serum chemistry** | | **BUN (mg/dL)** | **ALT (U/L)** | **ALP (U/L)** | **AST (U/L)** | **TBIL (mg/dL)** | **CA (mg/dL)** | **TP (g/dL)** | **ALB (g/dL)** | **NA+ (mmol/L)** |
| --- | --- | --- | --- | --- | --- | --- | --- | --- | --- | --- |
| Reference range  (C57BL/6) | Mean | 14 | 57 | 228 | 133 | 0.3 | 11.1 | 5.7 | 3.4 | 158 |
| 95% CI | High | 26 | 195 | 370 | 397 | 0.6 | 12.3 | 7.2 | 4.3 | 181.2 |
|  | Low | 5 | 27 | 105 | 43 | 0.2 | 9.7 | 4.8 | 2.4 | 147.5 |
|  | n | 159 | 158 | 160 | 161 | 156 | 155 | 159 | 157 | 66 |
| control | Mean | 21 | 21 | 110 | 70 | 0.3 | 9.9 | 4.7 | 3.9 | 146 |
|  | High | 24 | 23 | 119 | 75 | 0.4 | 10.2 | 4.7 | 4.0 | 148 |
|  | Low | 18 | 18 | 101 | 64 | 0.2 | 9.7 | 4.6 | 3.8 | 144 |
|  | n | 8 | 8 | 8 | 8 | 8 | 8 | 8 | 8 | 8 |
| PCOS | Mean | 18 | 43 | 102 | 101 | 0.3 | 10.3 | 4.7 | 4.0 | 147 |
|  | High | 19 | 62 | 110 | 147 | 0.4 | 10.5 | 4.9 | 4.2 | 148 |
|  | Low | 16 | 23 | 94 | 55 | 0.3 | 10.0 | 4.5 | 3.8 | 146 |
|  | n | 8 | 8 | 8 | 8 | 8 | 8 | 8 | 8 | 8 |
| PCOS+Dox | Mean | 22 | 66 | 76 | 144 | 0.4 | 12.9 | 4.7 | 3.9 | 149 |
|  | High | 29 | 120 | 84 | 209 | 0.4 | 13.8 | 4.8 | 4.1 | 151 |
|  | Low | 15 | 12 | 68 | 79 | 0.3 | 11.9 | 4.7 | 3.7 | 147 |
|  | n | 7 | 7 | 7 | 7 | 7 | 7 | 7 | 7 | 7 |

**Supplementary Figure S1**

Figure S1. Effect of doxercalciferol treatment on the expression of the immortalized adipocyte model (SW872) gene. (A) Altered gene expression in SW872 cells after doxercalciferol treatment (24 h). (B-E) Significantly downregulated genes in SW872 cells after doxercalciferol treatment. (F-H) Genes significantly upregulated in SW872 cells after doxercalciferol treatment. The data are presented as median and interquartile range (IQR) information to report non-normally distributed data. Unpaired T tests were performed for statistical comparisons between groups. (Significance level, *p < 0.05, **p < 0.005, ***p < 0.0005, ****p < 0.0001; NS: not significant.)

**Supplementary Figure S2**

Figure S2. Effect of doxercalciferol treatment on cytokine secretion in an immortalized adipocyte model (SW872). (A) Antibody-based obesity-related cytokine analysis of SW872 secretome data after doxercalciferol treatment (24h) (B-H) Cytokine production was significantly downregulated after treatment with doxercalciferol. The data are presented as median and interquartile range (IQR) information to report non-normally distributed data. Unpaired T tests were performed for statistical comparisons between groups. (Significance level, *p < 0.05, **p < 0.005, ***p < 0.0005, ****p < 0.0001; NS: not significant.)

**Supplementary Figure S3.**

Figure S3. Serum hormone analysis of PCOS mice

AMH levels were significantly greater in PCOS patients than in healthy controls. Doxercalciferol-treated mice still exhibited an increase in AMH levels, but this difference was not significant compared to that in the control group. Testosterone levels increased in the PCOS group but were not significantly different. Insulin levels did not change in any of the groups. The data are presented as median and interquartile range (IQR) information to report non-normally distributed data. One-way ANOVA were performed for statistical comparisons between groups. (Significance level, *p < 0.05, **p < 0.005, ***p < 0.0005, ****p < 0.0001; NS: not significant.)

**Supplementary Figure S4.**

Figure S4. Weekly-basis alpha diversity analysis of the fecal microbiome of PCOS mice.

On a weekly basis, the alpha diversity analysis did not reveal statistical significance due to the low sample number and high diversity.

**Supplementary Figure S5**

Figure S5. Weekly-based beta diversity analysis of the PCOS mouse fecal microbiome.

Weekly basis beta diversity analysis did not reveal statistical significance due to the low sample number and high diversity.
